# Cutaneous leishmaniasis on the malar region suggesting squamous cell carcinoma in two elderly patients^☆^

**DOI:** 10.1016/j.abd.2023.06.008

**Published:** 2024-02-24

**Authors:** Roberto Bueno Filho, Juliana Idalgo Feres, Natalia de Paula, Sebastião Antonio de Barros Júnior, Ana Maria Roselino

**Affiliations:** a Dermatology Division, Department of Medical Clinics, Faculdade de Medicina de Ribeirão Preto, Hospital Universitário, Universidade de São Paulo, Ribeirão Preto, SP, Brazil; b Laboratory of the Dermatology Division, Hospital Universitário da Faculdade de Medicina de Ribeirão Preto, Universidade de São Paulo, Ribeirão Preto, SP, Brazil; c Department of Pathology, Hospital Universitário da Faculdade de Medicina de Ribeirão Preto, Universidade de São Paulo, Ribeirão Preto, SP, Brazil

Dear Editor,

In Brazil, cutaneous leishmaniasis (CL) is prevalent among young males, who are more exposed to sandfly biting, but recently an increased number of CL in the elderly has been observed. CL presentation in elderly patients seems to be different due to decreased immunologic response that leads to larger lesions and mucosal involvement, longer disease duration, and less lymphadenopathy.^1^ CL in sun-exposed skin of the elderly can mimic squamous cell carcinoma (SCC).^2^, ^6^ Three men and one woman from 42- to 61-years-old were reported.^2^, ^3^, ^5^, ^6^ In one of them, CL diagnosis just followed Mohs surgery.^5^

We report two elderly patients presenting CL on their malar region whose initial histopathological exam diagnosed SCC for the first patient.

Case 1: A 83-year-old woman living in Pradópolis, São Paulo State, Brazil, presented a large ulcer with an infiltrative border on her right cheek for 4-months (Fig. 1A). Three other ulcers on her chin and tights, and a right supraclavicular lymph node were observed. She had chronic renal failure, arterial hypertension, and peripheral arterial stenosis. Histopathology of the cheek lesion showed pseudoepitheliomatous hyperplasia (PEH), atypical squamous cells and keratinous pearls that were reported first to an SCC diagnosis. A revision of the histopathological features showed a dermal granulomatous inflammatory infiltrate with the presence of plasm cells but the absence of amastigotes being compatible with leishmaniasis diagnosis (Fig. 1C‒D). The PCR followed by *Hae*III enzymatic restriction confirmed *Leishmania Viannia braziliensis* in a skin sample (for methodology).^7^ Montenegro skin test resulted in 3 × 4 mm of induration. Because liposomal amphotericin was not available, meglumine antimoniate 485 mg daily for 10 days, followed by amphotericin B 225 mg were prescribed, resulting in her cure (Fig. 1B). Unfortunately, both drugs were precociously withdrawn because of QT enlargement on electrocardiogram and atrial fibrillation, and progressive renal dysfunction. Regular follow-up showed no recurrence after 7 months.

**Figure 1:**
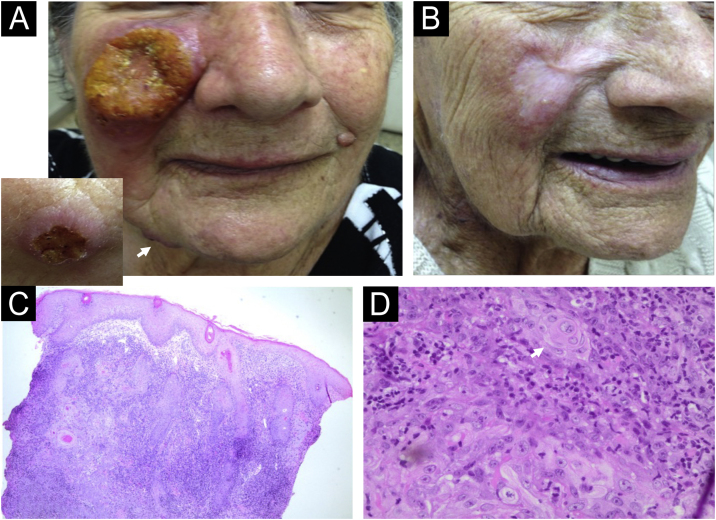
Case 1. (A) A large ulcer with infiltrative border on the erythematous-violaceous right cheek. The insert (left corner) shows the mandibular lesion (white arrow). (B) Complete remission of the lesions after meglumine antimoniate and amphotericin B treatment. (C) Histopathology of the malar ulcer biopsy showing pseudoepitheliomatous hyperplasia, and a huge granulomatous inflammatory infiltrate in the dermis (Hematoxylin & eosin, 4×). (D) Keratinous pearls (white arrow) and atypical squamous cells can be seen (Hematoxylin & eosin, 60×).

Case 2: A 73-year-old man living in Serra Azul, São Paulo state, Brazil, presented an infiltrative plaque of small ulcers and crusts on his right cheek lasting 20 days (Fig. 2A) without any lymphadenopathy. He had chronic renal failure, congestive heart failure, and prostatic cancer. Histopathology of the skin biopsy showed PEH, a dermal granulomatous inflammatory infiltrate with plasm cells, and rare amastigotes formed inside monocytes (Fig. 2C‒D). *Leishmania Viannia braziliensis* was identified by PCR-RT, corroborating CL diagnosis. Montenegro skin test resulted in 7 mm of induration. After improvement with 1.5 g of liposomal amphotericin, a persistent lesion on the scar border (Fig. 2B) was managed with one cycle of cryosurgery using an open probe. No recurrence after 6 years of follow-up was detected.

**Figure 2:**
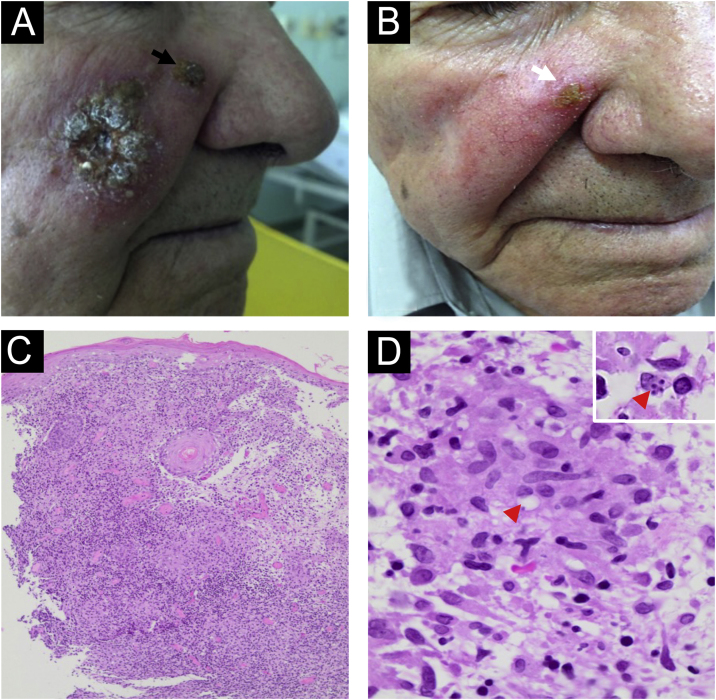
Case 2. (A) An infiltrative plaque of small ulcers and crusts in the right cheek, and a satellite lesion in the nasal border (black arrow). (B) The patient presented great improvement with 1.5 g of liposomal amphotericin. A lasted lesion (white arrow) was treated with cryosurgery using an open probe. (C) Histopathology showing pseudoepitheliomatous hyperplasia and a granulomatous infiltrate in the dermis (Hematoxylin & eosin, 4×). (D) Rare round structures inside macrophages suggesting amastigotes forms can be seen (red arrowhead) (Hematoxylin & eosin, 100×). The insert (right-up corner) shows four amastigotes inside a macrophage.

The clinical differential diagnosis of CL is challenging, as it can mimic infectious processes such as furuncles, ecthyma, tuberculosis, syphilis, leprosy, and deep fungal, and malignant skin tumors.^8^ Besides the clinical lesions of these two patients appearing in a sun-exposed area, the main confounding factor in the histopathological exam is the PEH, which can lead to a mistaken diagnosis of SCC.^6^ PEH results from chronic skin irritation and can be seen in the histopathological exams of all above differential diagnosis.^4^, ^6^, ^8^ Epithelial mitosis, keratin pearls, and PEH can be also seen in the histopathological exam of a CL biopsy lesion,^4^, ^6^ causing a misdiagnosed SCC hypothesis. When PEH is present, other features such as the cellular infiltrate must be considered in the differential histopathological description of CL and SCC.^6^

Laboratory exams are important to identify atypical and/or chronic cases of CL. However, a definitive etiological diagnosis is difficult to obtain since the parasite detection in the lesion is sometimes tricky.^9^ Here, both patients presented a positive epidemiology for tegumentary leishmaniasis, living in an endemic region, frequenting ranch, and having a fisher habit in riversides. Moreover, an undetermined/positive Montenegro skin test improved the clinical suspicion of CL. Of importance for etiological leishmaniasis diagnosis, the second patient presented amastigotes in his biopsy, and PCR confirmed *Leishmania Viannia braziliensis* in the two cases.^9^

Diagnosis and treatment of leishmaniasis are challenging in the elderly due to specific health characteristics: immune system impairment, hormonal changes, negligence to illness and treatment, irregular and multiple drug consumption, comorbidities, and atypical disease presentations.^1^ The woman patient presented four lesions and regional adenomegaly, and both patients had comorbidities, and particularities seen in the elderly. Liposomal amphotericin is the first drug preconized by the Brazilian Health Ministry for CL treatment in over 50 year old patients.^10^ Fortunately, both patients responded well to the treatment.

Some other aspects can be mentioned: the description of SCC in CL scars, the association of SCC and CL in the same tissue sample, and the parasite leishmania has been discussed as a promoter of cancer in immunocompromised hosts.^11^

In conclusion, the description of two cases of CL in sun-exposed skin of elderly patients draws attention to the specialists who deal with CL and SCC patients.

## Financial support

None declared.

## Authors' contributions

Ana Maria Roselino: Design and planning of the study; data collection and analysis and interpretation of data; drafting and editing of the manuscript and critical review of intellectual content; intellectual participation in the propaedeutic and/or therapeutic conduct; effective participation in research orientation; critical review of the literature and approval of the final version of the manuscript.

Roberto Bueno-Filho: Data collection and analysis and interpretation of data; drafting and editing of the manuscript and critical review of intellectual content; intellectual participation in the propaedeutic and/or therapeutic conduct; critical review of the literature and approval of the final version of the manuscript.

Juliana Idalgo Feres: Data collection and analysis and interpretation of data; drafting and editing of the manuscript and critical review of intellectual content; critical review of the literature and approval of the final version of the manuscript.

Natália de Paula: Data collection and analysis and interpretation of data; approval of the final version of the manuscript.

Sebastião Antonio de Barros Júnior: Data collection and analysis and interpretation of data; approval of the final version of the manuscript.

## Conflicts of interest

None declared.
